# Anatomic distribution of basivertebral foramen with a magistral form in vertebral bodies of T10~L5 and its clinical significance for extensive epidural cement leakage in cement-augmented pedicle screw fixation: a multicenter case–control study

**DOI:** 10.1186/s13018-023-04456-3

**Published:** 2024-01-04

**Authors:** Weibo Yu, Daozhang Cai, Zhensong Yao, Haiyan Zhang, Xiaobing Jiang

**Affiliations:** 1 Department of Spinal Surgery, The First Affiliated Hospital of Guangxi University of Chinese Medicine, Guangzhou, Guangdong People’s Republic of China; 2 Department of Spinal Surgery, The Second Affiliated Hospital of Guangzhou Medical University, Changgang East Road 250, Guangzhou, 510260 Guangdong People’s Republic of China; 3 Department of Orthopaedics, The Third Affiliated Hospital of Southern Medical University, Guangzhou, Guangdong People’s Republic of China; 4 Department of Spinal Surgery, The First Affiliated Hospital of Guangzhou University of Chinese Medicine, Guangzhou, Guangdong People’s Republic of China

**Keywords:** Cement-augmented pedicle screw fixation, Osteoporotic patients, Epidural cement leakage, Basivertebral foramen, Pedicle screw placement

## Abstract

**Background:**

There are no reports discussing anatomic distribution of basivertebral foramen (BVF) in the osteoporotic vertebral body, which is critical in the analysis of the risk of epidural cement leakage (ECL) after cement-augmented pedicle screw fixation (CAPSF).

**Methods:**

371 osteoporotic patients using 1898 cement-augmented screws were included. Preoperative computed tomography (CT) was used to determine the frequency, width, height, and depth of magistral BVF in T10~L5. Additionally, we measured the distance between BVF and the left/right borders of vertebral body as well as the distance between BVF and upper/lower endplates. Following CAPSF, the severity of ECL and the position of pedicle screws were determined by postoperative CT. Finally, significant risk factors for extensive ECL were identified through binary logistic regression analysis.

**Results:**

Of 2968 vertebral bodies ranging from T10 to L5, 801 (42.2%) had a magistral BVF. From T10 to L5, the frequency of magistral BVF appeared to gradually increase. The magistral BVF was much closer to the upper endplate and the depth accounted for about a quarter of anteroposterior diameter of vertebral body. Overall, there were 19 patients (5.1%) and 32 screws (1.7%) with extensive ECL, nine of whom had neurological symptoms. The independent risk factors for extensive ECL were the magistral BVF (OR = 8.62, *P* < 0.001), more volume of cement injected (OR = 1.57, *P* = 0.031), reduced distance from screw tip to vertebral midline (OR = 0.76, *P* = 0.003) and vertebral posterior wall (OR = 0.77, *P* < 0.001) respectively.

**Conclusion:**

When planning a CAPSF procedure, it is important to consider anatomical distribution of BVF and improve screw implantation methods.

## Introduction

The problems of pedicle screw loosening and pull-out have become well-known since the implementation of pedicle screw fixation in the management of degenerative conditions, fractures, tumors, and spinal deformities, particularly in individuals with osteoporosis [1, 2]. Consequently, spine surgeons have faced a difficult undertaking when attempting to implant a prosperous pedicle screw fixation in older individuals suffering from osteoporosis. Polymethylmethacrylate (PMMA) is a widely used method to enhance the strength of pedicle screws, and various studies, both in vitro and clinical, have demonstrated its significant effectiveness [1–5]. One major concern associated with this method is the potential for epidural cement leakage (ECL) [6–9]. The occurrence of such leaks is quite frequent and can result in either temporary or permanent impairment of neurological function. The occurrence rate of this complication differs based on the original state, like osteoporosis, and the amount of cement injected [7, 8].

In the augmentation of pedicle screws, cement leakage was categorized into three regions by Hu et al. [8]: anterior–lateral (affecting the front and/or front-side of the vertebral body), posterior–lateral (affecting the back-side of the vertebral body), and epidural leakage (affecting the spinal canal). Although leaks in the anterior–lateral and posterior–lateral regions were typically not deemed clinically significant, there have been reports indicating that epidural leakage carries a greater potential for causing neurological damage. A second assessment was conducted to determine the severity of ECL, as Bokov et al. [9] categorize it as either local or extensive. Certain factors that increase the risk of ECL have been discovered in CAPSF, such as reduced bone mineral density and the shape of cement distribution. However, the aforementioned study failed to examine the correlation between epidural leakage and additional potential risk factors, including the volume of cement injected per screw and the placement of the pedicle screw within the vertebral body, particularly in cases of extensive ECL. Moreover, our clinical practice has recently uncovered a potential risk for ECL after CAPSF in the form of a magistral basivertebral foramen (BVF) located in the middle section of the vertebral body's posterior wall. Hence, the primary objective of this investigation was to examine the anatomical distribution of BVF with a magistral form, additionally ascertain the factors associated with magistral ECL, and assess the potential impact of BVF with a magistral form on extensive ECL in osteoporotic individuals undergoing CAPSF treatment.

## Materials and methods

This study was designed to be performed retrospectively at three medical centers between January 2014 and May 2023. The research was conducted following the ethical guidelines outlined in the Declaration of Helsinki by the World Medical Association. Following the endorsement of the ethics committee at the main research organization, consent was acquired from either the director of the corresponding sites or the research ethical board at each local institution.

### Selection of patients

A total of 413 osteoporotic patients who underwent CAPSF at three medical centers were retrospectively reviewed. However, 42 patients were excluded due to incomplete clinical data (*n* = 24), spinal infection (*n* = 5), and spinal metastases (*n* = 13). Dual-energy X-ray absorptiometry was utilized to measure the bone mineral density (BMD) of every vertebral body. Osteoporosis was defined by setting the criteria as having a T-score value lower than − 2.5. In conclusion, a total of 371 individuals (54 males and 317 females) participated in the study. The average age of the participants was 66.29 ± 8.87 years. These individuals had T scores of -3.27 ± 0.94. Among them, 23 had osteoporotic vertebral fractures, 151 had lumbar spondylolisthesis, 77 had degenerative scoliosis, and 120 had lumbar spinal stenosis. To avoid biased effects, the cement-augmented pedicle screws located within the fractured vertebrae were excluded in our research. In the end, a total of 1898 pedicle screws were reinforced with cement. On average, the duration of an operation was 258.30 ± 61.31 min, with an average blood loss of 710.19 ± 575.66 ml, and an average hospital stay of 19.32 ± 7.41 days. Table 1 displayed comprehensive patient attributes. Before being included in the study, every participant provided their informed consent.

**Table 1:** Basic data of patients and augmented screws

*Clinical characteristics for the patient (n = 371)*	
Age (years)^a^	66.29 ± 8.87
Sex	
Male	54
Female	317
BMI^a^	22.67 ± 3.32
BMD (T score)^a^	− 3.27 ± 0.94
Diagnosis	
Osteoporotic vertebral fractures	23
Lumbar spondylolisthesis	151
Degenerative scoliosis	77
Lumbar spinal stenosis	120
No. of fused segments per patient	
Single segment	47
Double segments	164
Multiple segments	160
Blood loss (mL)^a^	710.19 ± 575.66
Operative time (mins)^a^	258.30 ± 61.31
Screw design	
Solid	101
Fenestrated	270
Epidural cement leakage	
No epidural leakage	301
Local epidural leakage	51
Extensive epidural leakage	19
*Characteristics for the augmented screws (n = 1898)*	
The level of augmented screws	
Thoracic segments (T10-T12)	47
Lumbar segments (L1-L5)	1851
Screw design	
Solid	428
Fenestrated	1470
Epidural cement leakage	
No epidural leakage	1754
Local epidural leakage	112
Extensive epidural leakage	32
The morphology of basivertebral foramen	
A magistral type	801
A dispersed type	1097
The position of pedicle screw in the vertebral body (mm)^a^	
The distance between screw tip and the midline of vertebral body	24.10 ± 3.25
The distance between screw tip and the posterior wall of vertebral body	5.47 ± 2.38
Cement extension into pedicle area	
Yes	110
No	1788

BMI body mass index, BMD bone mineral density

^a^Quantitative variables are expressed as mean ± SD

### Operative procedure

General anesthesia was used for all procedures. The patients were positioned in a prone stance, and the placement of screws was carried out following the method outlined in previous research studies [10–12]. The skilled surgeons determined whether to perform augmentation based on the assessment of the patients' BMD and the indication of inadequate mechanical strength of implanted screws found during tapping, as stated in previous studies [13, 14]. (1) Solid screw with PMMA prefilling: Using a typical vertebroplasty method, a firm screw with PMMA prefilling was employed to inject conventional PMMA cement (TECRES S.P.A, Sommacampagna, Italy) directly into the vertebral body through a 4-mm-diameter bone biopsy needle with live fluoroscopic guidance, in 0.1 ml increments. If the bone cement reaches the posterior vertebral body line or if there is any visible leakage of cement below the endplates or anterior cortex of the vertebral body, the injection of bone cement will be halted. Then, solid pedicle screws were quickly inserted after the biopsy needle was removed (Allegiance, Healthcare Co.). (2) Fenestrated pedicle screw with PMMA injection: A freehand insertion of fenestrated screws (RS8 LONG Minimally Invasive Spine System, REACH Medical, China) with PMMA injection was carried out on the spine affected by osteoporosis. The screws for the pedicle had a distal end that contained five holes and a cannulation diameter of either 2.5 mm or 3.0 mm. After confirming the screw position, the cement application system was securely attached to the top of the screw. Subsequently, PMMA cement (TECRES S.P.A, Sommacampagna, Italy) was injected in 0.1 ml increments with the guidance of lateral fluoroscopy. At each of the three medical centers, the process of PMMA preparation and application adhered to a standardized protocol that was supplied by the company. Hence, the time taken to combine the powder and liquid was 30 s, filling the application device also took 30 s, and it was necessary to wait for 300 s before using the cement. Each pedicle screw received an average cement injection of 2.15 mL, with a range of 0.6–5 mL. The operations were performed by three spinal surgeons with over 13 years of experience. The method does not necessitate a difficult learning process.

### Collection of data and evaluation using CT before and after surgery

**Fig. 1:**
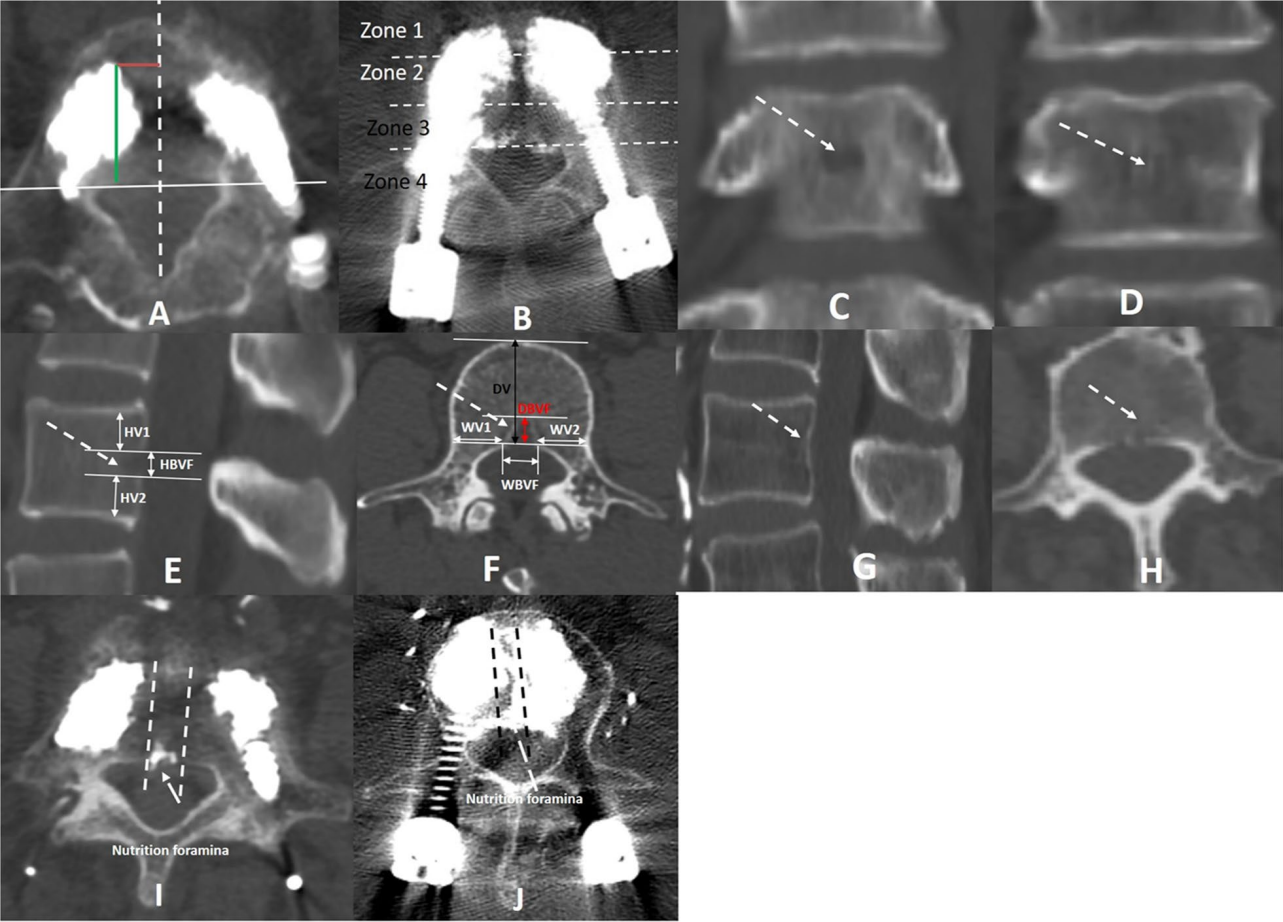
**A** On an axial CT scan, the axial location of pedicle screw implantation in the medio-lateral direction (indicated by the red line) and the anterior–posterior direction (indicated by the green line) within the vertebral body. **B** On CT axial view, the distribution of cement within the vertebral body was classified into four zones. **C**–**F**. A magistral form of basivertebral foramen (a large, single centrally located foramen with a width/height of ≥ 5 mm). **D**–**H**. A disperse form of basivertebral foramen (multiple small nutrient foramina). **I**. Local ECL on axial CT image (cement meniscus only at the nutrition foramen). **J**. Extensive ECL on axial CT image (cement mass surpassing the limits of the nutrition foramen in the posterior wall of the vertebral body, or leakage in the canal measuring over 2 mm in thickness). ECL epidural cement leakage

Every enrolled patient went through a non-contrast CT scan (Siemens SOMATOM PLUS 4, Germany) with a tube current of 240 mA, a KVP of 120, and a Pitch of 1 on both the day before surgery and the day after the surgery. The scans used a slice thickness of 0.5 mm and covered a scan area measuring 50 cm. Recorded were the imaging features, which encompassed the occurrence and anatomic distribution of BVF with a magistral form, the metrics related to the positioning of pedicle screws such as the screw tip's distances from the midline and posterior wall of the vertebral body, and cement extension within the vertebral body. Additionally, the cement extension within the vertebral body was categorized into four zones: Zone 1 represented the front third, zone 2 represented the middle third, zone 3 represented the back third, and zone 4 represented the pedicle area (Fig. 1A–B). Basivertebral foramina were categorized into two forms, depending on whether the posterior nutrition foramina were magistral (a large, centrally located foramen with a width/height of ≥ 5 mm) or dispersed (consisting of multiple small nutrient foramina), as shown in Fig. 1C–H. In conclusion, the seriousness of ECL was additionally noted and categorized into two sets: set 1, limited epidural leakage (cement meniscus solely at the nutrition foramen); set 2, extensive epidural leakage (cement mass surpassing the extent of the nutrition foramen in the posterior wall of vertebral body, or canal leakage measuring over 2 mm thick) (Fig.1I–J) [8, 9, 15, 16]. Two experienced spinal surgeons with more than a decade of expertise would independently measure all the radiological parameters twice in a double-blind manner. If there is a difference of opinion, a third radiologist with expertise is included in the decision-making process to address any bias.

### Statistical analysis

The statistical analyses in this study were conducted using SPSS 25.0 (SPSS, Inc., Chicago, IL, USA) and R Software version 3.6.2. Notable disparities were observed when the p value was less than 0.05. Qualitative characteristics of groups were expressed using means and standard deviations (SD). We evaluated the distinctions among the groups by employing univariate analysis for variables with symmetrical distributions and the nonparametric Wilcoxon test for variables with different distributions. To evaluate the statistical significance of binary variables, Fisher's exact test was employed. We employed binary logistic regression analysis and ROC curves to identify independent risk factors, respectively.

## Results

### The frequency and anatomic distribution of BVF with a magistral form in CT images of T10~L5

**Fig. 2:**
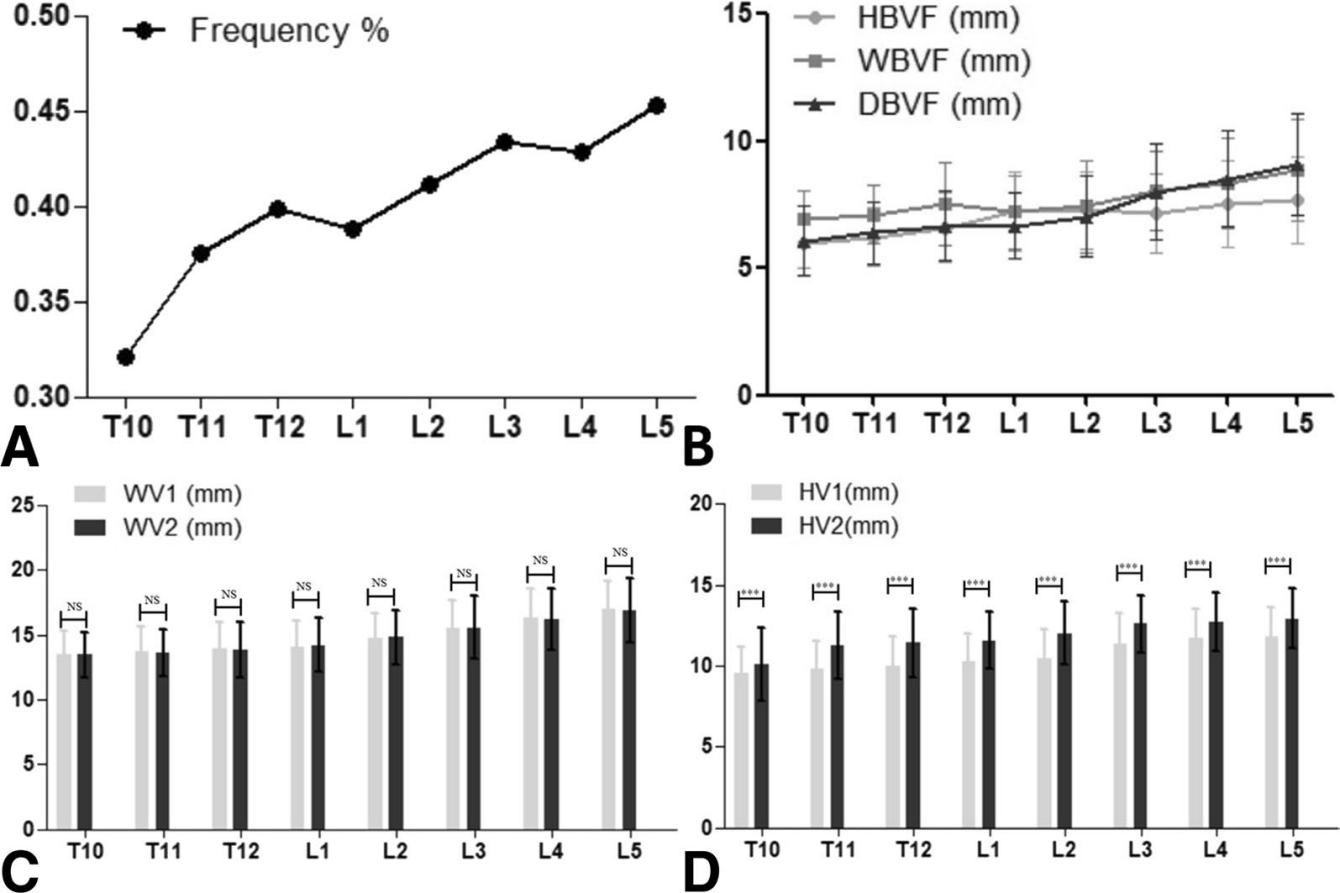
The frequency and different parameters of the basivertebral foramen with a magistral form in different levels from T10 to L5. **A** The frequency of the basivertebral foramen in different levels. **B** The height, width and depth of  the basivertebral foramen in different levels. **C** The distance between the basivertebral foramen and the upper/lower endplates of vertebral body. **D** The distance between the basivertebral foramen and left/right borders of vertebral body. HBVF the height of basivertebral foramen, HV1 the distance between basivertebral foramen and left borders of vertebral body, HV2 the distance between basivertebral foramen and right borders of vertebral body, WBVF the width of basivertebral foramen, WV1 the distance between basivertebral foramen and upper endplates of vertebral body, WV2 the distance between basivertebral foramen and lower endplates of vertebral body, DBVF the depth of basivertebral foramen

Out of a total of 2968 vertebral bodies spanning from T10 to L5, 1181 (39.8%) exhibited a magistral form of BVF. The prevalence of BVF with a magistral form was discovered to differ based on the vertebral level. In general, there was a consistent increase from T10 to L5, as shown in Table 2 and Fig. 2A). Similarly, the size of BVF with a magistral form including the width, height, and depth also gradually increased from T10 to L5 (Table 2 and Fig. 2B). Generally, the breadth of BFV represented approximately 25% of the front-to-back measurement of the vertebral body (0.24 ± 0.05), and there were no notable variances among various levels of the spine (*P* < 0.05) (Table 2).

**Table 2:** The measurement results for anatomic distribution of basivertebral foramen with a magistral type in CT images of T10~L5

	T10	T11	T12	L1	L2	L3	L4	L5
Frequency % (n)	32.1% (119)	37.5% (139)	39.9% (148)	38.8% (144)	41.2% (153)	43.4% (161)	42.9% (159)	45.3% (168)
HBVF (mm)	5.99 ± 0.98	6.23 ± 1.12	6.59 ± 1.36	7.24 ± 1.53	7.28 ± 1.52	7.19 ± 1.56	7.54 ± 1.71	7.66 ± 1.69
HV1(mm)	9.54 ± 1.66	9.81 ± 1.75	10.03 ± 1.79	10.23 ± 1.77	10.42 ± 1.81	11.39 ± 1.82	11.71 ± 1.76	11.81 ± 1.82
HV2 (mm)	10.10 ± 2.28	11.26 ± 2.04	11.40 ± 2.11	11.57 ± 1.73	11.99 ± 1.95	12.57 ± 1.80	12.71 ± 1.80	12.92 ± 1.84
WBVF (mm)	6.95 ± 1.07	7.12 ± 1.14	7.54 ± 1.65	7.20 ± 1.45	7.43 ± 1.79	8.05 ± 1.53	8.38 ± 1.78	8.86 ± 2.01
WV1 (mm)	13.48 ± 1.81	13.75 ± 1.92	13.91 ± 2.03	14.12 ± 1.93	14.73 ± 1.94	15.51 ± 2.22	16.33 ± 2.27	16.96 ± 2.18
WV2 (mm)	13.48 ± 1.74	13.66 ± 1.80	13.83 ± 2.13	14.20 ± 2.09	14.83 ± 2.10	15.59 ± 2.40	16.24 ± 2.38	16.87 ± 2.45
DBVF (mm)	6.07 ± 1.35	6.39 ± 1.25	6.67 ± 1.38	6.65 ± 1.29	7.04 ± 1.58	8.01 ± 1.86	8.53 ± 1.91	9.07 ± 1.99
DV (mm)	26.26 ± 1.79	27.34 ± 1.42	27.98 ± 1.53	28.07 ± 1.68	29.33 ± 2.02	32.13 ± 2.81	32.34 ± 2.25	32.19 ± 2.33
DBVF/DV	0.23 ± 0.04	0.23 ± 0.04	0.24 ± 0.05	0.24 ± 0.05	0.23 ± 0.05	0.26 ± 0.05	0.26 ± 0.05	0.27 ± 0.05

HBVF the height of basivertebral foramen, HV1 the distance between basivertebral foramen and left borders of vertebral body, HV2 the distance between basivertebral foramen and right borders of vertebral body, WBVF the width of basivertebral foramen, WV1 the distance between basivertebral foramen and upper endplates of vertebral body, WV2 the distance between basivertebral foramen and lower endplates of vertebral body, DBVF the depth of basivertebral foramen, DV the anteroposterior diameter of vertebral body

No significant difference was observed between the left and right borders of the vertebral body at different vertebral levels when measuring the distance between BVF and the left/right borders (Table 2 and Fig. 2C). In addition, we assessed the distance between the BVF and the upper and lower endplates of the vertebral body. Our findings revealed that the BVF was notably nearer to the upper endplate compared to the lower endplate (situated at the plane of the vertebral pedicle's inferior margin) across all T10-L5 vertebral levels (*P* < 0.05) (Table 2 and Fig. 2D).

### Characteristics of patients and their clinical results

**Fig. 3:**
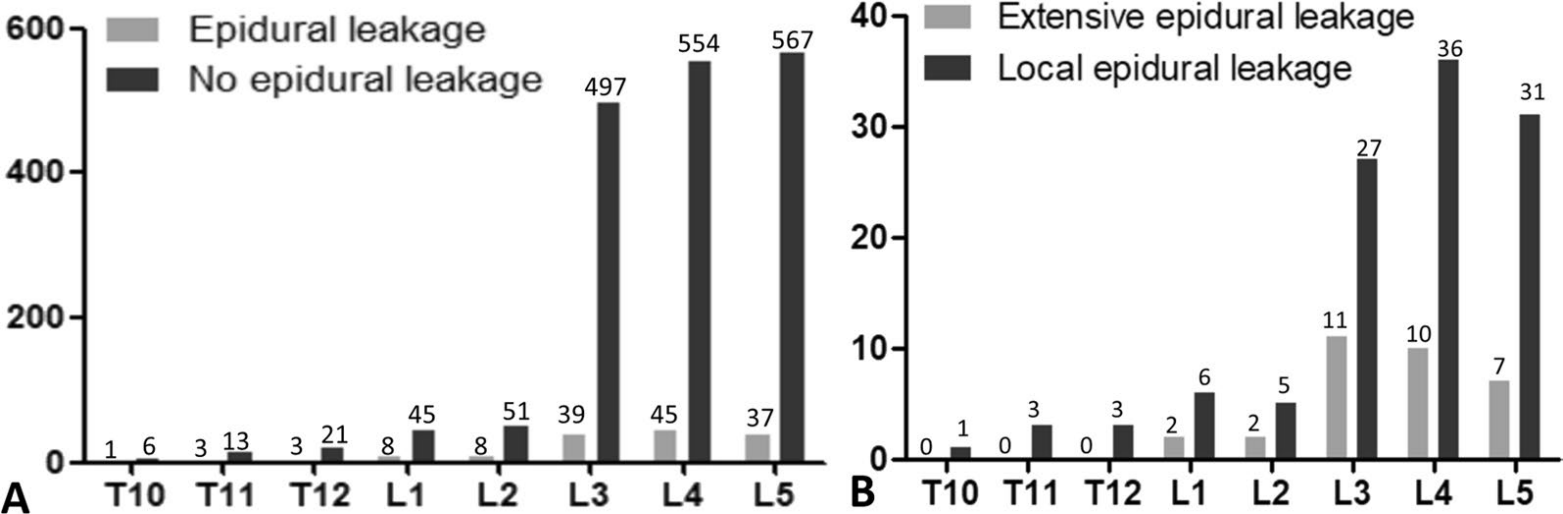
The arrangement of pedicle screws in relation to ECL. **A** Distribution of pedicle screws with and without ECL **B** Distribution of pedicle screws with different forms of ECL. ECL epidural cement leakage

ECL occurred in 70 out of 371 patients (18.9%), with the involvement of 144 out of 1898 augmented screws (7.6%). The levels treated were from T10 to S1, and Fig. 3 illustrates the distribution of pedicle screws and ECL.

Nineteen patients were found to have extensive ECL, with a total of 32 screws involved. Out of these patients, ten were asymptomatic and had 13 screws affected. Out of the 19 patients, nine individuals (with 19 screws) developed neurological symptoms resulting from nerve compression caused by ECL. Among these, seven patients experienced mild sciatica after the surgery but recovered with conservative treatment. Two patients suffered neurological injury and needed a second surgery for total laminectomy at the L1/L2 level on the second day after the operation. However, even after 1 year of follow-up, they still reported weakness and numbness in their right leg. A total of 51 patients were found to have local ECL, which affected 112 screws. It is worth noting that none of the patients experienced any symptoms.

Out of the 1898 augmented screws, a significant number of 801 (42.2%) exhibited a magistral form of BVF, with 26 (3.2%) showing extensive ECL. Out of the 1898 augmented screws, a scattered form of BVF was noticed in 1097 (57.8%), with only 6 (0.5%) showing extensive ECL. Statistical significance of these variations was determined through the application of Fisher's exact test, yielding a P value of less than 0.001.

### Binary logistic regression analysis and ROC curve analysis were used to determine independent risk factors

For augmented screws with extensive ECL, a univariate analysis (Table 3) indicated that five factors were significantly linked to a higher risk of developing extensive ECL. These factors include the form of BVF (*P* < 0.001), injected cement volume (*P* = 0.004), screw tip distance from the vertebral body midline (*P* = 0.004), screw tip distance from the vertebral body posterior wall (*P* < 0.001), and cement extension into the pedicle area (*P* = 0.016).

**Table 3:** Univariate analysis results for augmented screws with extensive epidural cement leakage

	Extensive epidural Leakage (*n* = 32)	Non-extensive epidural Leakage (*n* = 1866)	T/χ^2^/Z	*P* values
The type of basivertebral foramen (magistral/dispersed)	26/6	775/1091	20.346	< 0.001*
Volume of cement injected (ml)^a^	2.56 ± 0.83	2.16 ± 0.78	− 2.876	0.004*
The distance between screw tip and the midline of vertebral body (mm)^a^	4.27 ± 1.57	5.49 ± 2.39	2.894	0.004*
The distance between screw tip and the posterior wall of the vertebral body (mm)^a^	21.56 ± 3.58	24.15 ± 3.22	4.491	< 0.001*
Cement extension into pedicle area (yes/no)	5/27	105/1761	5.760	0.016*
Screw design (solid/fenestrated)	10/22	418/1448	1.411	0.235

Bold values indiacte statistically significant difference

^a^Quantitative variables are expressed as mean ± SD

*P < 0.05

**Fig. 4:**
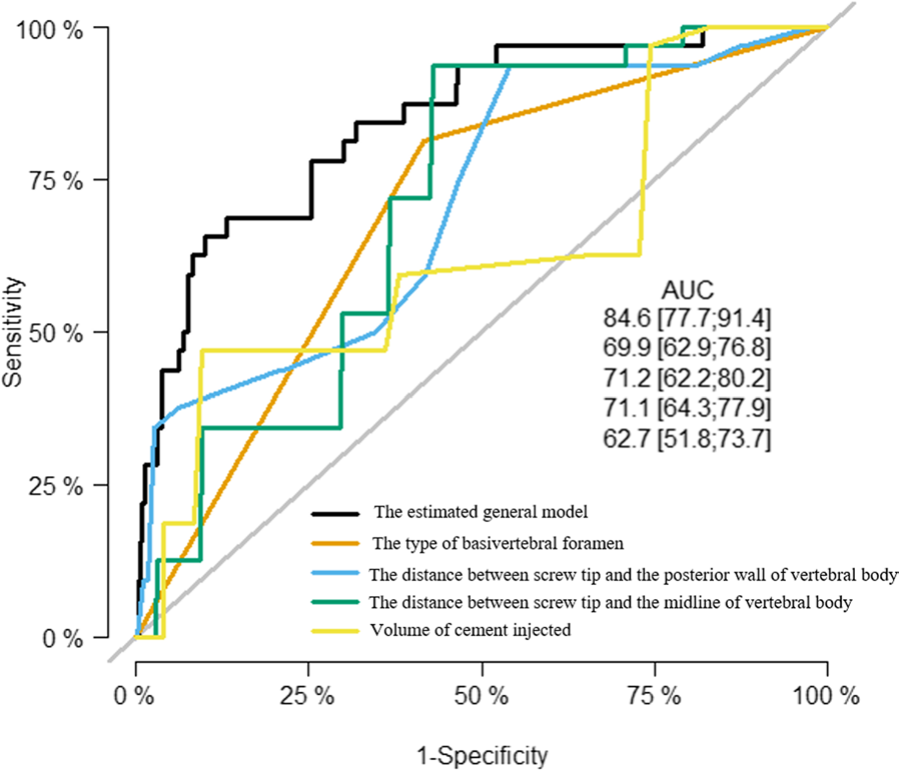
The estimated general model and each independent risk factor for augmented screws with extensive epidural cement leakage were evaluated using the receiver operating characteristic (ROC) curve and area under the curve

Additionally, Table 4 presented the application of multivariate logistic regression to augmented screws with extensive ECL. Four independent risk factors were identified, including a magistral form of BVF (OR = 8.62, 95% CI 3.44–21.59, *P* < 0.001), increased volume of injected cement (OR = 1.57, 95% CI 1.04–2.37, *P* = 0.031), reduced distance between screw tip and vertebral body midline (OR = 0.76, 95% CI 0.64–0.91, *P* = 0.003), and decreased distance between screw tip and vertebral body posterior wall (OR = 0.77, 95% CI 0.69–0.85, *P* < 0.001). Comparing with each individual risk factor, the AUC of the estimated overall model for extensive ECL was 0.846 (95% CI 0.777 to 0.914), exhibiting the highest sensitivity of 65.6% and specificity of 90.1% (Fig. 4).The Hosmer–Lemeshow *p* value of 0.633 further confirmed the satisfactory calibration of the estimated regression model.

**Table 4:** Using multivariate logistic regression analysis to judge independent risk factors for augmented screws with extensive epidural cement leakage

	Regression coefficients (B)	OR	95% confidence interval of OR		*P* values
			Lower bound	Upper bound	
The type of basivertebral foramen (magistral/dispersed)	2.154	8.616	3.438	21.591	< 0.001
The distance between screw tip and the midline of vertebral body(mm)^a^	− 0.269	0.764	0.639	0.913	0.003
The distance between screw tip and the posterior wall of the vertebral body (mm)^a^	− 0.264	0.768	0.692	0.852	< 0.001
Volume of cement injected (ml)^a^	0.452	1.572	1.043	2.370	0.031

OR odd ratio

^a^Quantitative variables are expressed as mean ± SD

## Discussion

The scarcity of comparative studies between various vertebral levels is widely recognized in the literature concerning the anatomic distribution of posterior cortical BVF. Li et al. [17] conducted a previous investigation using cadaver specimens and found that the BVF was positioned next to the middle section of the back wall of the vertebral body, and the microstructural characteristics of the trabecular bone in the area surrounding the BVF were comparatively reduced compared to the upper or lower regions. Nevertheless, the anatomic distribution of BVF with a magistral form, along with its frequency and size, had not been specifically addressed by them. In our current research, we observed a gradual rise in the occurrence of BVF with a magistral form from T10 to L5. The BVF with a magistral form was much closer to the upper endplate and the depth accounted for about a quarter of anteroposterior diameter of vertebral body.

Leakage of cement into the spinal canal is a frequently encountered complication associated with CAPSF and carries an increased likelihood of developing neurological impairments [1–4]. Regrettably, fluoroscopic guidance is unable to prevent or detect the majority of ECL cases, as the postoperative CT results are significantly greater than those identified during intraoperative fluoroscopy [18]. Hence, it is crucial to possess a comprehensive comprehension of the possible hazards associated with ECL. Until now, previous research has only discovered a limited number of possible hazards linked to factors related to the procedure, such as the placement of the screw tip in the mediolateral direction of the vertebral body and the distribution of cement within the vertebral body. However, it is unclear whether and how natural anatomical and morphological differences in posterior cortical BVF affect ECL during CAPSF in depth studies. Moreover, it remains uncertain if the screw depth in the front-back orientation of the vertebral body has a significant correlation with ECL while performing CAPSF.

According to Hu et al. [6] and Georgy et al. [19], ECL is categorized based on its thickness. It remains uncertain whether this grading system holds any clinical significance. In conclusion, our research concentrated on extensive ECL because of the potential for neurological complications linked to this form of leakage. The analysis of binary logistic regression revealed that extensive ECL was significantly associated with four factors: increased amount of cement injected, reduced distance between screw tip and the midline of vertebral body, and decreased distance between screw tip and the posterior wall of the vertebral body.

Our study revealed that an increase in the amount of cement injected was associated with a higher probability of extensive ECL. Previous research has indicated that it also posed a potential danger of epidural leakage in vertebroplasty or kyphoplasty [20, 21]. Injecting a significant quantity of bone cement may lead to elevated injection pressure, potentially resulting in the rupture of secondary venous walls and the intrusion of cement into the spinal canal through the BVF. Previous studies have suggested that the quantity of cement per screw should range from 1.8 to 3.0 ml [7, 10]. The purchasing strength is not increased by injecting more cement than 2.8 ml per screw, according to biomechanical studies. In order to minimize the possibility of epidural leakage, we consider utilizing a quantity of 1.5–3.0 ml for each pedicle screw as an appropriate measure [10].

Moreover, the positioning of pedicle screws also exhibited a notable correlation with substantial ECL. Instances where the screw tip depth was reduced or the screw tip was in closer proximity to the midline were found to increase the likelihood of extensive ECL. Additionally, the structure of the BVF in the posterior cortex has been identified as another important factor in clinical settings. A magistral form of the BVF has shown a strong correlation with a significantly increased likelihood of extensive ECL. It is our belief that the anatomical features of the basivertebral vein could potentially have a significant impact on the underlying cause. It starts in the lower third of the spinal column and merges toward the back to empty into the front section of the internal venous network. If screws are inserted too shallow or near the center, a larger amount of cement injected can easily enter the main basivertebral system and be transferred to the front epidural space through these veins, potentially affecting the neural components in the spinal canal [7, 22, 23].

Based on our discoveries, we concluded that it was clinically important to analyze the anatomical dispersion of BVF with a magistral form in order to avoid excessive ECL during the planning of a CAFPSF operation. To be away from the magistral form of BVF, it is recommended to implant pedicle screws in the middle upper axis of the pedicle, ensuring a safe distance from the vertebral body's inferior margin plane where the foramen is primarily located. Meanwhile, during cement injection, the injection pressure should not be excessive and it should be performed under serial lateral fluoroscopic guidance. Since the magnitude of BFV with a magistral form constituted approximately 25% of the front-to-back measurement of the vertebral body (0.24 ± 0.05), we deemed it imperative to cease the injection of bone cement when the front portion of the vertebral body was mostly occupied.

The current study still has certain limitations. Initially, we implemented a multicenter retrospective clinical study; however, it is unavoidable to encounter selection bias and biases related to technical and interpretational aspects among surgeons. Consequently, further verification and evaluation of a prospective, cadaveric experimental study would be justifiable. Secondly, our study did not measure the viscosity of bone cement. It was reported that higher viscosity helped prevent epidural leakage and lower viscosity led to extravasation. The viscosity of cement may be influenced by factors such as storage conditions, mixing techniques, and the temperature of the operating room [7, 8, 24], although there was no universally accepted benchmark. Therefore, as part of the study's standard CAFPSF procedure, we suggest using toothpaste consistency of cement (after blending powder and liquid, with a waiting period of 390 s), gradually injecting it and verifying distribution consecutively through fluoroscopy. Furthermore, in our investigation, most of the pedicle screw tip's location could be determined either directly or by modifying the window level and width on post-operative CT scans. Nevertheless, a minor fraction of the screw tip's location remained concealed, prompting us to ascertain the screw tip's position by relying on the measurements of screws implanted during the operation. Despite the possibility of certain bias compared to directly detecting the position of the screw tip, we are confident that the ultimate outcomes would not be substantial. Additionally, epidural leakage may be influenced by the presence of various fenestrated pedicle screws that have different core diameters, sizes, numbers, and radial hole placements [25, 26]. To avoid biased effects from various forms of fenestrated pedicle screws, our study exclusively focused on a single form of fenestrated pedicle screw.

## Conclusion

The occurrence of BVF with a magistral form seemed to progressively rise from T10 to L5. A magistral form of BVF was in much closer proximity to the upper endplate, with its depth representing approximately 25% of the anteroposterior diameter of the vertebral body. ECL is quite prevalent in patients with osteoporosis who are undergoing CAFPSF treatment. Although local ECL is typically not of clinical importance, the presence of extensive ECL increases the likelihood of experiencing neurological symptoms. When planning a CAFPSF procedure, it is important to consider the structure of the BVF. Improvement of screw implantation techniques is crucial, along with ensuring that cement injection volumes do not exceed the anterior three-fourths of the vertebral body, particularly in cases with a magistral BVF.

## Data Availability

This study work was not supported by any funding sources.

## Abbreviations

CAPSF: Cement-augmented pedicle screw fixation
ECL: Epidural cement leakage
BVF: Basivertebral foramen
BMI: Body mass index
BMD: Bone mineral density
HBVF: The height of basivertebral foramen
HV1: The distance between basivertebral foramen and left borders of vertebral body
HV2: The distance between basivertebral foramen and right borders of vertebral body
WBVF: The width of basivertebral foramen
WV1: The distance between basivertebral foramen and upper endplates of vertebral body
WV2: The distance between basivertebral foramen and lower endplates of vertebral body
DBVF: The depth of basivertebral foramen
DV: The anteroposterior diameter of vertebral body
PMMA: Polymethylmethacrylate
ROC: Receiver operation characteristic
OR: Odds ratios

## Availability of data and materials

Data are available upon reasonable request.
